# Global research states and trends of micro RNA in irritable bowel syndrome: a bibliometric analysis

**DOI:** 10.1007/s10238-024-01396-y

**Published:** 2024-07-05

**Authors:** Hongxiu Chen, Zhifang Xu, Honggang Zhao, Jiazhen Cao, Rui Wang, Jing He, Ru Nie, Jialin Jia, Shuting Yuan, Yonghong Li, Zhicheng Liu, Xinyu Zhang, Lijuan Ha, Xiaoru Xu, Tie Li

**Affiliations:** 1 College of Acupuncture and Tuina, Changchun University of Chinese Medicine, No. 1035, Boshuo Rd, Jingyue Economic Development District, Changchun, 130117 People’s Republic of China; 2 National Clinical Research Center for Chinese Medicine Acupuncture and Moxibustion, No. 10, Poyang Lake Road, West District, Tuanbo New Town, Jinghai District, Tianjin, 301617 People’s Republic of China; 3 Research Center of Experimental Acupuncture Science, Tianjin University of Traditional Chinese Medicine, No. 10, Poyang Lake Road, West District, Tuanbo New Town, Jinghai District, Tianjin, 301617 People’s Republic of China; 4 Shenzhen Hospital of Integrated Chinese and Western Medicine, 528 Xinsha Road, Shajing Street, Baoan District, Shenzhen, People’s Republic of China

**Keywords:** Irritable bowel syndrome, Non-coding RNA, Global research, Bibliometric, microRNA

## Abstract

Irritable bowel syndrome (IBS) is a common chronic gastrointestinal disorder, but its diagnosis and treatment remain obscure. Non-coding RNAs (ncRNAs), as potential biomarkers, have attracted increasing attention in digestive diseases. Here, we present a comprehensive research status, development trends, and valuable insights in this subject area. The literature search was performed using Web of Science Core Collection. VOSviewer 1.6.20, Citespace 6.2.R4, and Microsoft Excel 2021 were used for bibliometric analysis. A total of 124 articles were included in the analysis. Overall, publication patterns fluctuated. Globally, People’s Republic of China, the USA, and Germany were the top three contributors of publications. Guangzhou University of Chinese Medicine, University of California, Mayo Clinic, and University of California, Los Angeles contributed the highest number of publications. The pathways and specific mechanisms by which ncRNAs regulate transcription and translation and thus regulate the pathophysiological processes of IBS are the main research hotspots in this field. We found that microRNA (miRNAs) are intricately involved in the regulation of key pathologies such as viscera sensitivity, intestinal permeability, intestinal mucosal barrier, immunoinflammatory response, and brain-gut axis in the IBS, and these topics have garnered significant attention in research community. Notably, microecological disorders are also associated with IBS pathogenesis, and ncRNA may play an important role in the interactions between host and intestinal flora. This is the first bibliometric study to comprehensively summarize the research hotspots and trends related to IBS and ncRNAs (especially miRNAs). Our findings will help understand the role of ncRNAs in IBS and provide guidance to future studies.

**Supplementary Information:**

The online version contains supplementary material available at 10.1007/s10238-024-01396-y.

## Introduction

Irritable bowel syndrome (IBS) is a common functional gastrointestinal disorder characterized by recurrent abdominal pain with changes in fecal matter or bowel habits and has recently been defined as a disorder of the bowel–brain interaction [1, 2]. This is because a large proportion of patients with IBS experiences extraintestinal symptoms, including psychological, psychiatric, and mood disorders [3–5]. With the changing times, the burden of life and work has increased, and the diets and rest patterns of individuals have become extremely irregular, which can easily induce IBS. A recent international cross-sectional study reported that the overall prevalence of IBS in East Asia was 12.6% [6]. Although IBS is not fatal, it seriously affects health and quality of life and imposes a heavy financial burden on families and social healthcare [7]. The pathogenesis of IBS is complex and involves visceral hypersensitivity (VH) [8], intestinal flora imbalance [9], reduced immune activation [10], an overactive serotonergic (5-HT) system [11], and intestinal barrier dysfunction [12]. The diagnosis of IBS has no precise criteria, but based on Rome IV [13] and Bristol Stool Form, it is classified into four subgroups: diarrhea-predominant IBS (IBS-D), constipation-predominant IBS (IBS-C), IBS with mixed defecation habits (IBS-M), and unclassified IBS (IBS-U) [14]. However, these criteria are highly subjective and do not accurately differentiate between patients with IBS and healthy individuals. Therefore, developing biomarkers for the accurate diagnosis of IBS is important.

The current clinical practice for IBS primarily focuses on symptom management. Although symptomatic medications can significantly relieve symptoms in the short term, their long-term efficacy is not outstanding [15]. Some researchers have suggested that actively understanding the epigenetic mechanisms underlying IBS may help develop more durable and effective treatments. Fortunately, epigenetic modifications, including chromatin remodeling, DNA methylation, and non-coding RNA (ncRNA), have been identified in the occurrence and development of IBS [16], among which ncRNA is a significant factor in gene expression. Studies on ncRNA correlations based on IBS pathophysiology have proven invaluable in uncovering disease mechanisms and exploring diagnostic and treatment approaches. ncRNA, which does not code for proteins but has enzymatic, structural, or regulatory functions, is involved in a variety of biological processes, including gene regulation, RNA modification, and other cellular processes. According to their length and functional strength, ncRNAs can be divided into different categories. Among these, microRNA (miRNA) and long non-coding RNA (lncRNA) have been widely studied [17].

Bibliometric analysis can quantitatively describe and visualize the key features of published literature in a certain field and has been widely used in various disciplines and fields [18–20]. It helps understand the current status of research in a field by comparing and analyzing different authors, institutions, countries, journals, and cited literature. In addition, visual analysis of keywords can help us deeply understand research hotspots and predict future development trends. This approach plays an important role in quickly and accurately understanding the research content in a field. Studies on the global trend of intestinal microbiota [21], VH [22], brain-gut axis [23], and IBS [24] have been conducted, and the status and development of the subfield of IBS have been evaluated, which has important guiding significance for further research on IBS. However, no hotspot or trend-tracking of ncRNA and IBS research has been conducted to date. This study aimed to comprehensively and systematically review the current status of research on ncRNA-related IBS worldwide to compensate for the bibliometric analysis deficiency and to help clinicians have a better understanding and reference for future research in this field.

## Methods

### Data source and search strategies

**Fig. 1:**
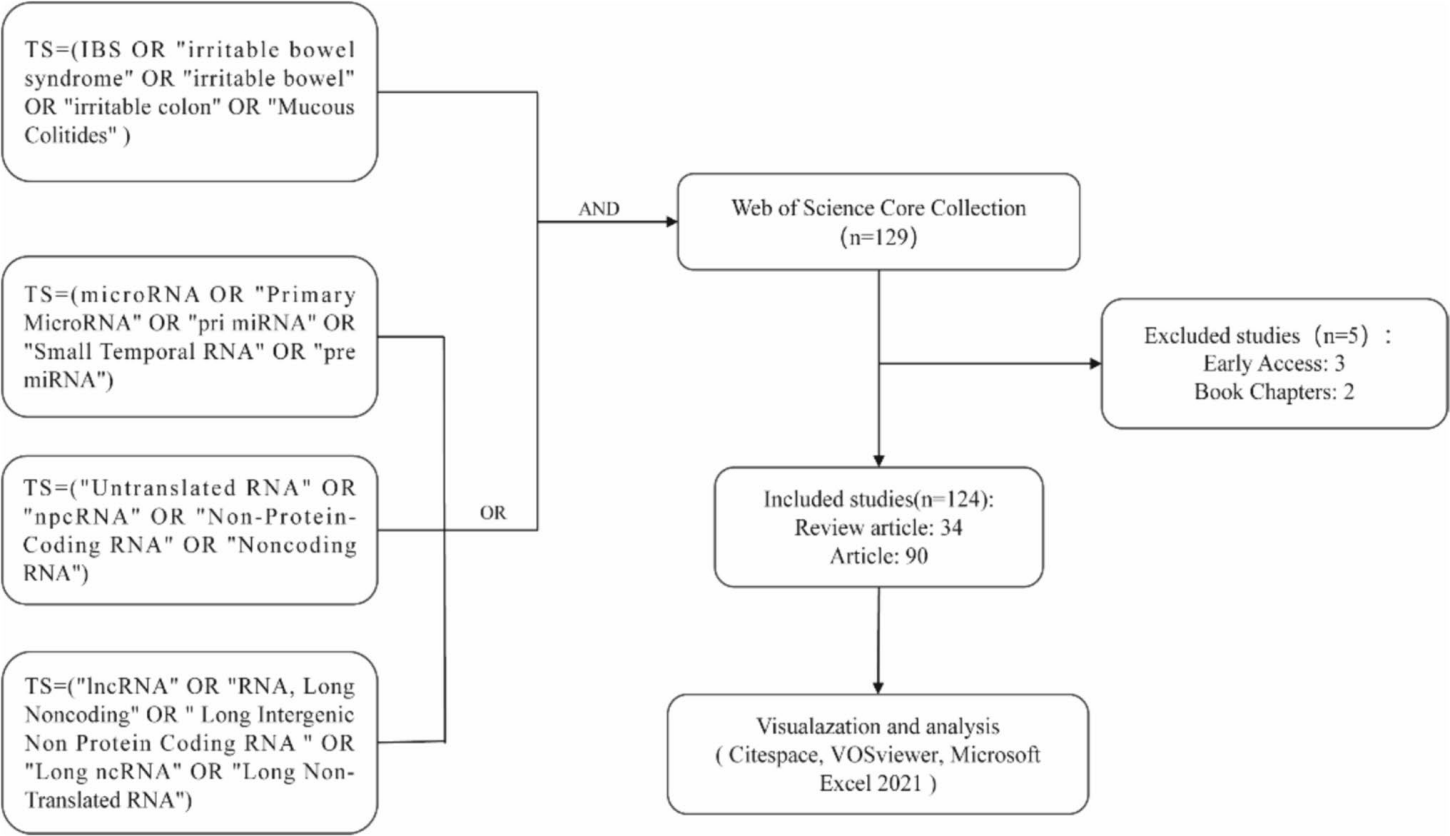
Flow diagram of the included articles

All publications were collected from the Core Collection database of the Web of Science (WoS) (http://apps.webofknowledge.com), which is the most authoritative database of scientific publications in a wide range of research areas. The search terms included (“Irritable Bowel Syndrome” OR “IBS” OR “irritable bowel” OR “irritable colon” OR “Mucous Colitides”) AND (“Untranslated RNA” OR “Non-coding RNA” OR “microRNA” OR “Primary MicroRNA” OR “RNA, Long Non-coding” OR “LincRNAs” OR “Small Temporal RNA”). All electronic searches were performed on January 11, 2024. Document types were limited to original research articles and reviews. Data were exported as “plain text files—full records with cited references” and saved in the “download_*” format. The detailed retrieval results are presented in Fig. 1.

### Data acquisition and processing

Data for this study were obtained within 1 day to eliminate potential discrepancies due to daily database updates. Before formal visualization of the analysis, literature was read and screened, duplicates and literature not relevant to the study were eliminated, and criteria were standardized for terms with the same meaning, such as (“Irritable Bowel Syndrome” and “IBS”) and (“microRNA” and “miRNA”). Studies with a high correlation between ncRNAs and IBS were screened, and basic article information (including title, author, and publication year), subjects, tissues, and the expression of ncRNAs and their targets in the pathological state of IBS were extracted. VOSviewer 1.6.20 and CiteSpace 6.2. R4 were used to identify and analyze the top-ranked countries, institutions, authors, journals, keywords, and co-cited literature. Additionally, Microsoft Excel 2021 (Microsoft Corporation, Redmond, WA, USA) was used. The data in this study were obtained directly from secondary-level databases and analyzed on this basis; thus, no ethical approval was obtained.

### Data analysis

Each dot in the visualization map represents an individual. The larger the dot, the higher the index of the relevant target. The line between the dots represents the correlation, and the thickness of the line represents correlation strength; the thicker the line, the higher the correlation degree. The contents of the studies were automatically grouped and color-coded, and the number of clusters varied according to the similarity threshold between the nodes. CiteSpace node Settings: Years per slice:1, TOP N:50, node types select country, institution, author, and keyword as visual objects, and perform the corresponding cluster analysis and emergence analysis for keywords. The keyword-pruning item selects Pathfinder, prunes the sliced networks, and prunes the merged network. Microsoft Excel 2021 was used to publish the trend statistics, sort the data, and generate the related tables.

## Results

### Annual publications and trends

**Fig. 2:**
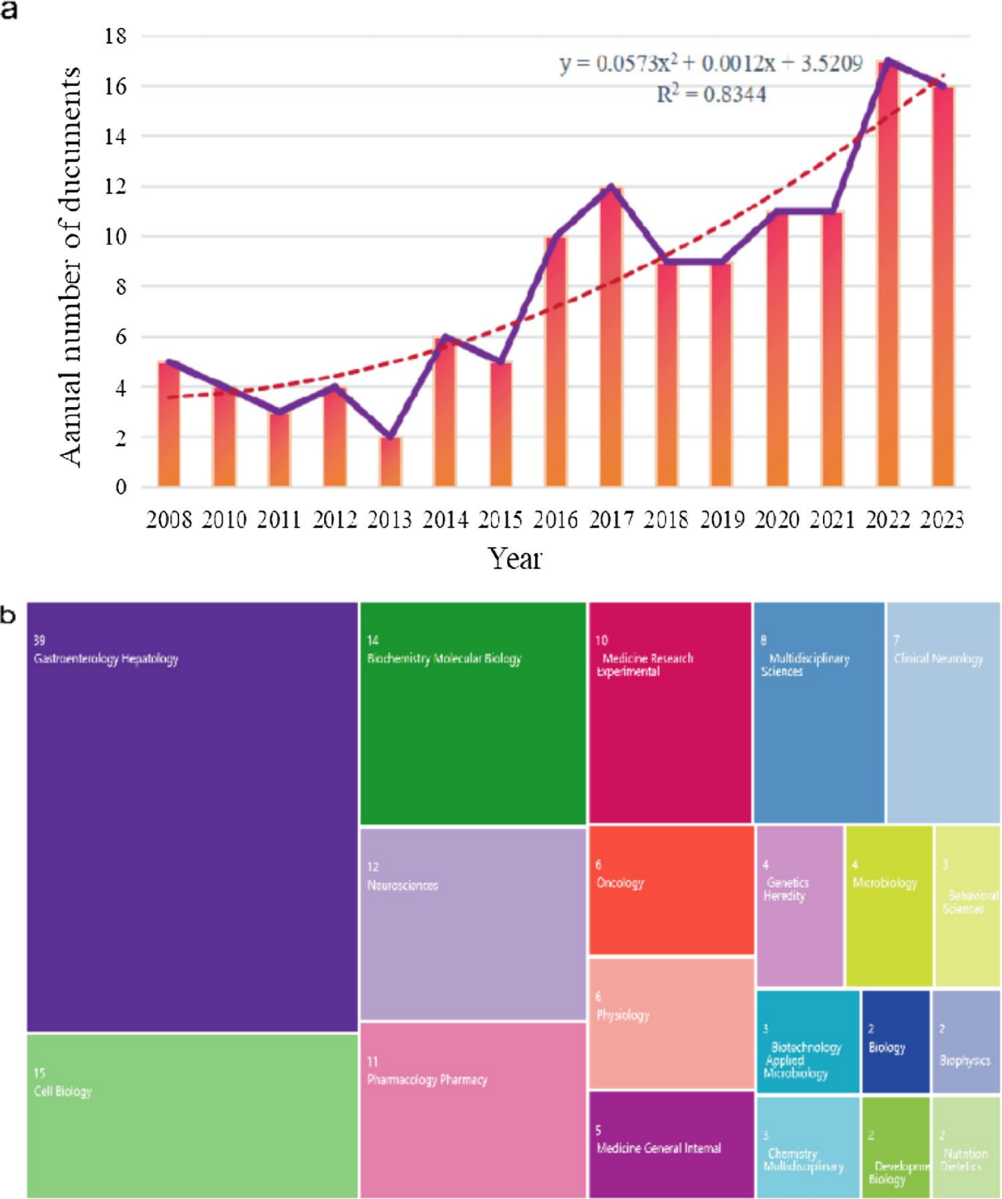
Global characteristics of articles published on WOS: **a** annual publication volume of articles and **b** WoS catalog classification of the research area

Ninety articles and 34 reviews met the Web of Science Core Collection retrieval criteria. The number of published studies is an important indicator of the development of a research field and helps us understand the field’s focus and predict future trends. As shown in Fig. 2a, since the first relevant study was published in 2008, the overall research output on ncRNAs in the field of IBS has shown a steady upward trend, with a trend line equation of *y* = 0.0573*x*^2^ + 0.0012*x* + 3.5209 (*R*^2^ = 0.8344), where *y* represents the study volume and *x* represents the corresponding year of publication. The polynomial fitting simulation curves exhibited a positive trend in this field. Figure 2b shows the catalog classification of Web of Science (WoS) distribution domains involved in this study, covering multidisciplinary areas, such as gastroenterology hepatology, cell biology, and biochemistry molecular biology.

### Analysis of countries and institutions

**Fig. 3:**
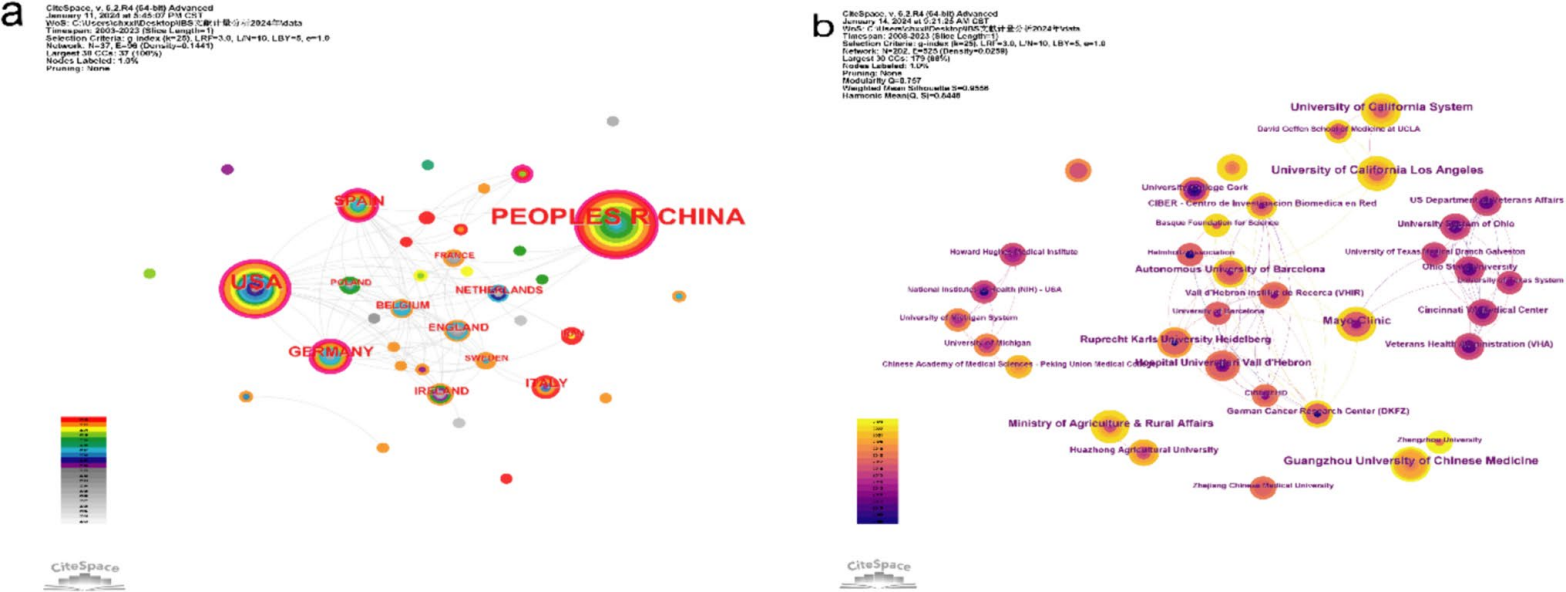
Networks of cooperative relationships of countries/institutions: **a** country partnership network and **b** institutional partnership network

The analysis of country/institution cooperation helps us objectively identify the relevant institutions concerned with research in a field and the cooperation between them to understand their influence on the development of a discipline. In Fig. 3a, each circle represents a country, circle size is proportional to the number of published articles in that country, and line thickness indicates the connections between different countries. The thicker the line, the more connections there are, i.e., the closer the degree of cooperation. China (0.17), USA (0.25), and Germany (0.3) were the top three countries in ncRNAs and IBS research, and they maintained active collaboration with each other and with other countries. Table 1 shows the top 10 countries according to the number of publications and centrality, and it can be seen that the number of publications and centrality is not the same. In CiteSpace, nodes with intermediate centrality greater than 0.1 are key nodes connecting other countries, representing collaboration, indicating an important core hub position in the research area. Germany (0.3), Norway (0.29), USA (0.25), and Spain (0.2) are the top four countries with the highest centrality.

**Table 1:** Countries/institutions ranked by publications and centrality

Items	Ranking	Publications	Country/Institution	Centrality	Country/Institution
Country	1	55	PEOPLES R CHINA	0.3	Germany
	2	35	USA	0.29	Norway
	3	10	GERMANY	0.25	USA
	4	10	SPAIN	0.2	Spain
	5	7	ITALY	0.17	Peoples R China
	6	5	ENGLAND	0.09	Netherlands
	7	5	IRELAND	0.08	IRELAND
	8	4	BELGIUM	0.08	INDIA
	9	4	NETHERLANDS	0.05	ENGLAND
	10	3	FRANCE	0.02	Belgium
Institution	1	7	Guangzhou University of Chinese Medicine	0.09	Mayo Clinic
	2	7	University of California System	0.08	German Cancer Research Center (DKFZ)
	3	7	Mayo Clinic	0.06	Autonomous University of Barcelona
	4	7	University of California Los Angeles	0.05	University of California Los Angeles
	5	6	Ministry of Agriculture & Rural Affairs	0.05	Hospital Universitari Vall d'Hebron
	6	5	Hospital Universitari Vall d'Hebron	0.03	Cornell University
	7	5	Autonomous University of Barcelona	0.02	Ruprecht Karls University Heidelberg
	8	5	Ruprecht Karls University Heidelberg	0.02	CIBER—Centro de Investigacion Biomedica en Red
	9	4	University System of Ohio	0.02	University of Texas System
	10	4	Vall d'Hebron Institut de Recerca (VHIR)	0.02	University of Texas Medical Branch Galveston

Visualization analysis revealed that 202 institutions contributed to this field. A detailed list of the publications of representative institutions and their collaboration strengths is shown in Table 1. Mayo Clinic had the highest number of publications (*n* = 7; 5.6%) and centrality (0.09), with earlier publication and lower collaboration with other institutions, followed by Guangzhou University of Chinese Medicine, University of California System, and University of California Los Angeles in terms of publication number. Institutional partnerships are shown in Fig. 3b.

### Analysis of authors

**Fig. 4:**
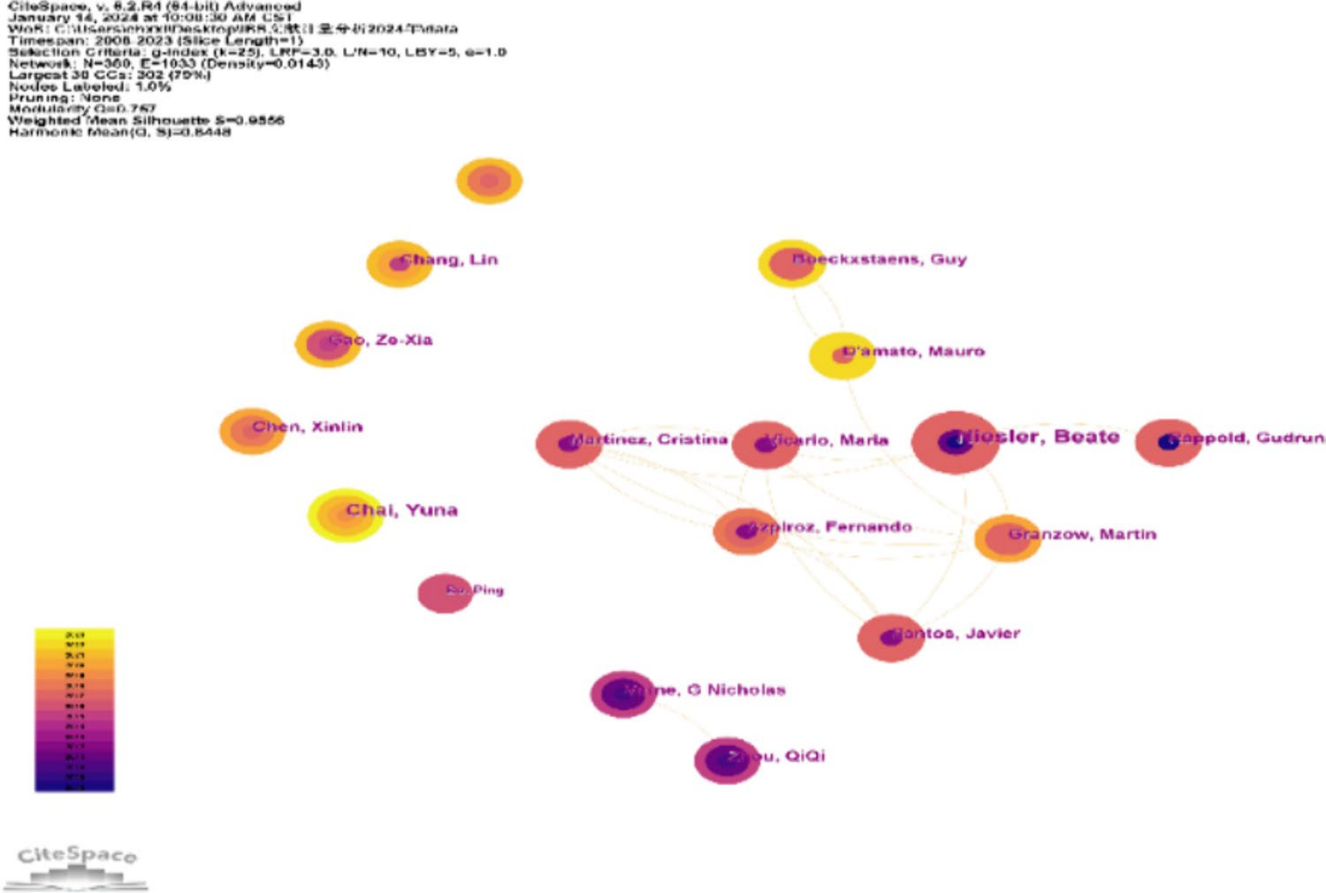
Author collaboration network

The authors’ co-map helps not only present influential research groups in the field but also uncover the nature of collaborative and cooperative teams. In addition, it helps researchers to identify potential collaborators. Figure 4 shows the author’s cooperative network diagram with 380 nodes and 1033 connections. The cooperation among researchers in different teams is evidently not close. The top seven authors and most representative articles are listed in Table 2. Niesler Beate, Professor of Human Molecular Genetics, University of Heidelberg, Germany, has made the largest contribution, with 325 cited publications. However, intermediate centrality among the authors was low.

**Table 2:** Authors ranked by publications and second by centrality

Ranking	Authors	Publications	Centrality	Totol citations	Selected publications (Highest citation)
1	Niesler, Beate	5	0.01	325	First evidence for an association of a functional variant in the microRNA-510 target site of the serotonin receptor-type 3E gene with diarrhea-predominant irritable bowel syndrome
2	Chai, Yuna	4	0	50	miRNA-29a modulates visceral hyperalgesia in irritable bowel syndrome by targeting HTR7
3	Granzow, Martin	3	0.02	134	miR-16 and miR-125b are involved in barrier function dysregulation through the modulation of claudin-2 and cingulin expression in the jejunum in IBS with diarrhoea
4	Boeckxstaens, Guy	3	0.01	135	Dietary and pharmacological treatment of abdominal pain in IBS
5	Martinez, Cristina	3	0.01	282	The Jejunum of Diarrhea-Predominant Irritable Bowel Syndrome Shows Molecular Alterations in the Tight Junction Signaling Pathway That Are Associated With Mucosal Pathobiology and Clinical Manifestations
6	Azpiroz, Fernando	3	0.01	258	The Jejunum of Diarrhea-Predominant Irritable Bowel Syndrome Shows Molecular Alterations in the Tight Junction Signaling Pathway That Are Associated With Mucosal Pathobiology and Clinical Manifestations
7	D'amato, Mauro	3	0.01	42	miR-16 and miR-103 impact 5-HT4 receptor signaling and correlate with symptom profile in irritable bowel syndrome

## Research hotspots and trends

### Analysis of keywords, clusters, timeline, and burst terms

Keywords represent the research focus and hotspots in the field. Table 3 shows the frequency and centrality of the top 23 ncRNA keywords in IBS research. “Irritable bowel syndrome” has the highest frequency, followed by “expression” and “genes.” In addition, keywords such as “inflammation,” “association,” “microRNAs,” “Crohn’s disease,” “cerebral palsy,” “cells,” “cancer,” and “disease” appear more than 10 times, revealing the current hotspots in this field. Centrality and frequency were not entirely consistent. As shown in Table 3, “genes” (0.44) had the highest centrality, followed by “cells” (0.3), “disease” (0.3), “association” (0.29), “gut microbiota” (0.28), “diarrhea” (0.28), “predominant irritable bowel syndrome” (0.23), “Crohn’s disease” (0.21), “disorders “(0.21), and “16 s ribosomal RNA” (0.2). These keywords play key roles in studying the correlation between ncRNAs and IBS. Notably, bidirectional regulation between the microbiota and host is a new direction for IBS and ncRNA research.

**Table 3:** Top 23 keywords in terms of frequency and centrality

Rank	Keywords	Frequency	Keywords	Centrality
1	Irritable bowel syndrome	73	Genes	0.44
2	Expression	42	Cells	0.3
3	Genes	16	Disease	0.3
4	Inflammation	14	Association	0.29
5	Association	13	Gut microbiota	0.28
6	Micrornas	12	Diarrhea-predominant irritable bowel syndrome	0.23
7	Crohns disease	12	Crohns disease	0.21
8	Cells	12	Disorders	0.21
9	Cancer	10	16 s ribosomal rna	0.2
10	Disease	10	Expression	0.19
11	Colorectal cancer	9	Irritable bowel syndrome	0.16
12	Gut microbiota	8	Brain	0.14
13	Pain	8	In vivo	0.14
14	Visceral hypersensitivity	8	Down regulation	0.13
15	Gene expression	7	Diarrhea	0.13
16	Barrier function	7	Colorectal cancer	0.12
17	Ulcerative colitis	7	Barrier function	0.1
18	Disorders	7	Identification	0.1
19	Activation	7	Animal models	0.1
20	Identification	6	Inflammation	0.09
21	Permeability	6	Fecal microbiota	0.09
22	Abdominal pain	6	Biomarkers	0.09
23	Intestinal permeability	6	Epithelial cells	0.09

**Fig. 5:**
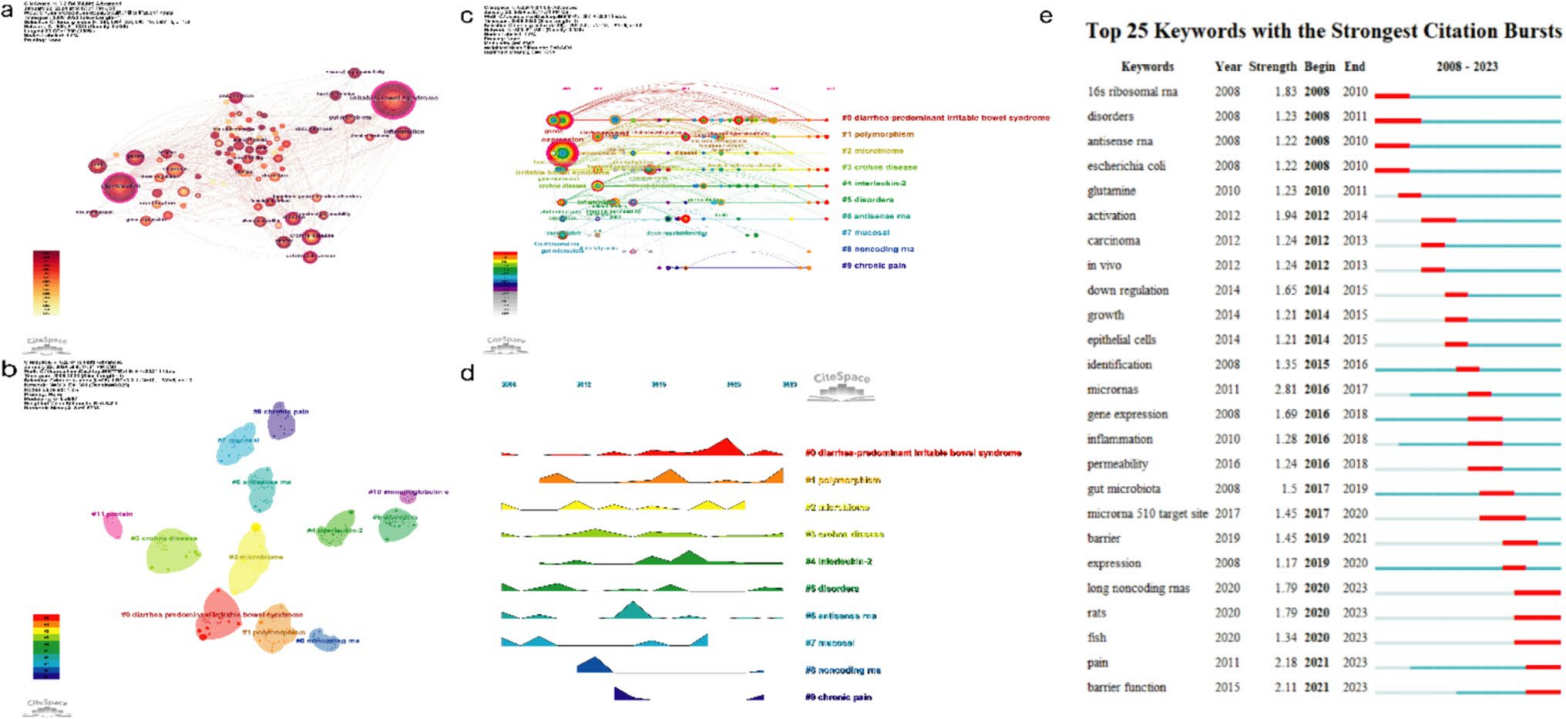
Keyword analysis network: **a** co-occurring keyword network, **b** co-occurring keyword-clustering network based on LLR algorithm, **c** timeline of co-cited clusters, **d** landscape of co-cited clusters, and **e** the top 17 keywords with the strongest citation bursts

Clustering and analyzing keywords can reveal research trends and development directions in a field. The keywords were clustered in CiteSpace. When Q > 0.3, the clustering structure is significant, and S > 0.5 indicates that the clustering results are reasonable [25]. Here, Q = 0.5667 and S = 0.8491. The log-likelihood ratio (LLR) algorithm was selected for keyword clustering, and 15 clusters were formed; the top 10 clusters are shown in Fig. 5b. #0 and #3 were associated with IBS, ulcerative colitis (UC), colorectal cancer, and dysplasia. This is because the onset of IBS often overlaps with other functional gastrointestinal diseases. #2, #4, #5, #7, and #9 were mainly related to the pathophysiological mechanisms of IBS, including visceral pain, intestinal permeability, mucosal structure, interleukin (IL), lymphocyte-associated inflammation–immunity interactions, and microbiota. The keyword-clustering labels and other keywords included are listed in detail in Table 4.

**Table 4:** Keywords cluster analysis

Cluster	Size	Sihouette	Mean Year	Label (LLR)	Other keywords
#0	39	0.866	2017	Diarrhea-predominant irritable bowel syndromes	Expression; development molecular mechanism; microrna-29a
#1	37	0.836	2017	Polymorphism	Psychiatry; HTR3e; variant database; non-celiac gluten sensitivity
#2	34	0.909	2015	Microbiome	Randomized controlled trial; functional gastrointestinal disorders; DNA methylation; histone modifications
#3	33	0.791	2015	Crohns disease	Ulcerative colitis; IBD; colorectal cancer; dysplasia
#4	29	0.692	2017	Interleukin-2	Life stress; interleukin-6; tumor necrosis factor-alpha; lymphocytes
#5	29	0.82	2013	Disorders	Abdominal pain; glutamine; visceral pain; intestinal permeability
#6	27	0.798	2013	Antisense RNA	Non-coding RNAs; escherichia coli; system; mouse model
#7	26	0.929	2013	Mucosal	mRNA; histamine; dietary supplements; short-chain fatty acids
#8	19	0.954	2013	Non-coding RNA	Transposon; RNA catalysis; splicing; RNA structure

The timeline and landscape displays of the keywords are shown in Fig. 5c and d. ncRNAs, especially miRNA expression in IBS, have been the focus of research, and understanding the structure and function of the corresponding ncRNA can help identify potential biomarkers with diagnostic and therapeutic value. Since the promulgation of Rome IV in 2016, more studies have focused on IBS subtypes, particularly IBS-D. Distinguishing the different subtypes of IBS helps to formulate targeted clinical treatment plans and achieve optimal efficacy.

Furthermore, keywords were analyzed by year to show the development trends in this field. The top 25 keywords with the strongest citation bursts are shown in Fig. 5e. The length of the red line represents the duration of the keyword burst. Since 2016, the most cited keywords in this field were “microRNAs,” “gene expression,” “inflammation,” “gut microbiota,” “barrier function,” “pain,” “long non-coding RNAs,” etc. The main research direction represented by these keywords was based on the differential expression and function of ncRNAs in the pathological mechanisms of IBS. “microRNAs” (2.81) had the highest burst intensity, followed by “pain” (2.18), “barrier function” (2.11), and “long non-coding RNAs” (1.79). These keywords emerged in 2020 and represent the future development trends.

### Analysis of high co-cited references

**Fig. 6:**
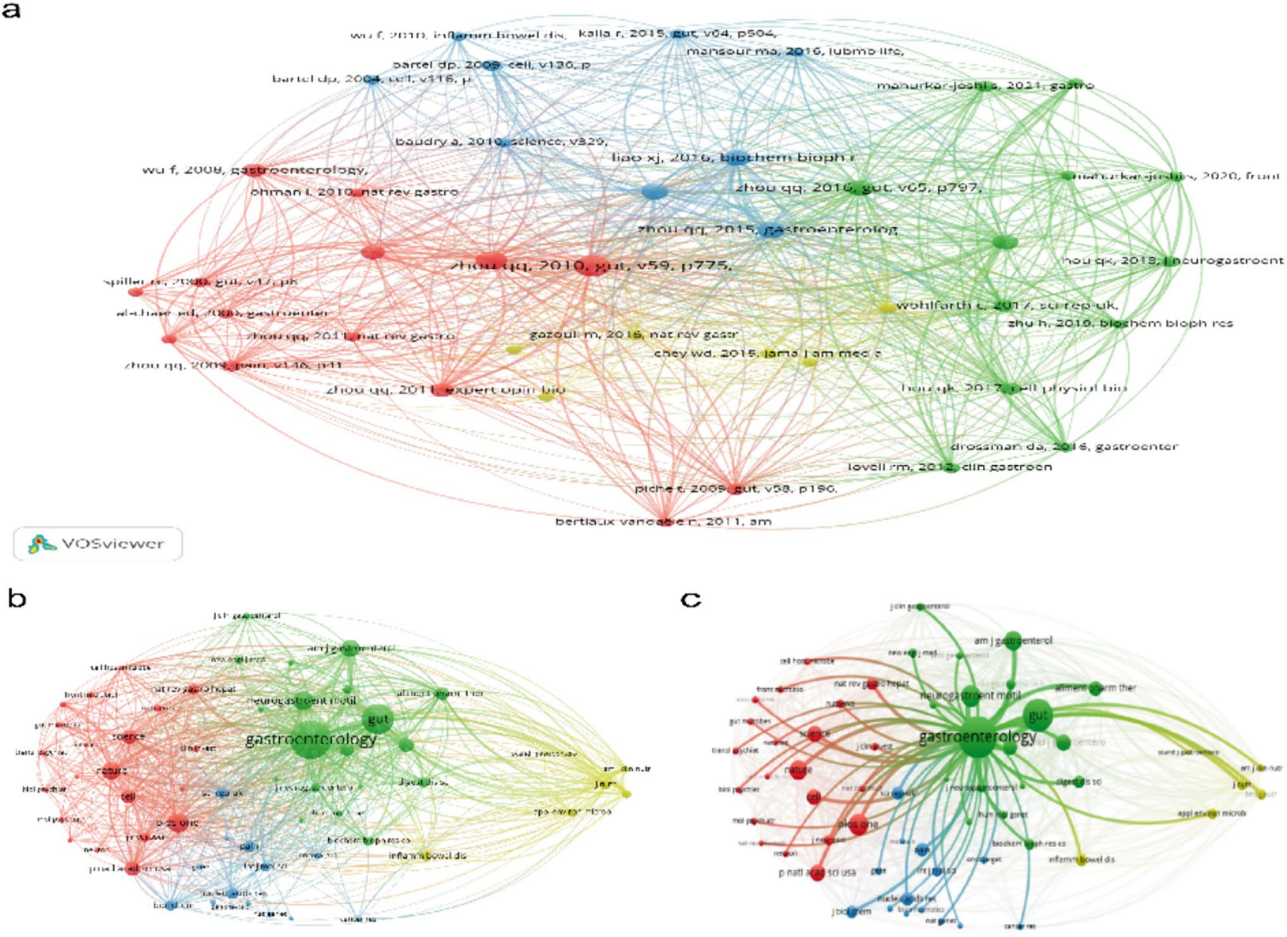
Cluster map of co-cited references and journals: **a** cluster map of co-cited references, **b** cluster map of co-cited journals, and **c** the highest co-cited journal

Reference co-cited analysis can efficiently and quickly locate key knowledge information in a field from a large number of references and visually show the strength of the correlation in literature, which is an important indicator for quantifying and reflecting the academic influence of references. Vosview’s results showed that, among the 7,562 co-cited literature, 41 studies had co-citation frequencies > 7. Figure 6a shows the network relationship and correlation strength. As shown in Fig. 6a, co-cited literature was categorized into four clusters. The red cluster is related to the pathophysiological mechanism of IBS, intestinal permeability, visceral sensitivity, and inflammation-immune-neural interactions, including increased expression of endocrine cells and T lymphocytes [26] in PI-IBS, downregulation of tight junction (TJ) proteins ZO-1, occludin, and claudin-1 in IBS-D [27–29], as well as VH associated with dysfunction of the 5-HT system [27]. The green cluster focuses on the interactions between ncRNAs and IBS and emphasizes the important role of epigenetic mechanisms in IBS. For example, miR-200a inhibits cannabinoid receptor 1 and 5-HT transporters, thus increasing visceral sensitivity in IBS-D rats [30]. Furthermore, miR-144 increases intestinal permeability in IBS-D rats by upregulating occludin and ZO-1 [31]. The blue cluster tends to elaborate on the emerging functions of miRNAs in IBS, demonstrating that miRNAs may become potential biomarkers for IBS diagnosis and treatment. The yellow cluster indicates IBS research progress. The top 10 articles by total citation frequency and correlation intensity ranked from high to low are shown in Table 5. “MicroRNA-29a regulates intestinal membrane permeability in patients with irritable bowel syndrome” is a widely cited paper published in *Gut* in 2010 [32], and the research group’s research on miR-29 has been continuing. In 2015, the same authors published a study in *Gastroenterology* reporting that miR-29 targets and reduces the expression of claudin-1 and nuclear factor-κb (NF-κB) repressing factors to increase intestinal permeability [33].

**Table 5:** Top 10 high co-cited reference ranked by citations

Ranking	Title	Citations	Years	Total link strength
1	MicroRNA-29a regulates intestinal membrane permeability in patients with irritable bowel syndrome	40	2010	424
2	First evidence for an association of a functional variant in the microRNA-510 target site of the serotonin receptor-type 3E gene with diarrhea-predominant irritable bowel syndrome	31	2008	343
3	MicroRNA 29 Targets Nuclear Factor-κB-Repressing Factor and Claudin 1 to Increase Intestinal Permeability	25	2015	316
4	Decreased miR-199 augments visceral pain in patients with IBS through translational upregulation of TRPV1	23	2016	295
5	Elevated circulating miR-150 and miR-342-3p in patients with irritable bowel syndrome	22	2014	266
6	MicroRNA-24 inhibits serotonin reuptake transporter expression and aggravates irritable bowel syndrome	22	2016	281
7	miR-16 and miR-125b are involved in barrier function dysregulation through the modulation of claudin-2 and cingulin expression in the jejunum in IBS with diarrhoea	21	2017	270
8	Functional bowel disorders	18	2006	164
9	MicroRNAs Are Differentially Expressed in Ulcerative Colitis and Alter Expression of Macrophage Inflammatory Peptide-2α	16	2008	108
10	miRNA-based therapies for the irritable bowel syndrome	16	2011	142

### Analysis of high co-cited journals

Among the 1,846 journals, 12 with a total citation frequency > 90 were analyzed using VosView, as shown in Fig. 6b and c. The journal of *Gastroenterology* had the highest co-cited rate and association strength, which were strongly consistent with disease attributes. Research achievements in this field are mostly displayed in high-quality journals such as *Gut*, *Science*, and *Nature*, indicating the degree of attention paid to research in this field (Table 6).

**Table 6:** Top 10 high co-cited journals ranked by citations

Rank	Journal	Counts	Citations	Total link strength
1	Gastroenterology	95	507	11,371
2	Gut	89	356	8471
3	Neurogastroent Motil	62	183	5556
4	Plos one	67	182	4094
5	Nature	53	154	4486
6	Am J Gastroenterol	62	150	4176
7	P Natl Acad Sci Usa	55	124	3166
8	Science	51	115	3421
9	World J Gastroentero	53	111	2983
10	Cell	43	108	3165

## Interaction mechanisms of IBS and ncRNA

**Fig. 7:**
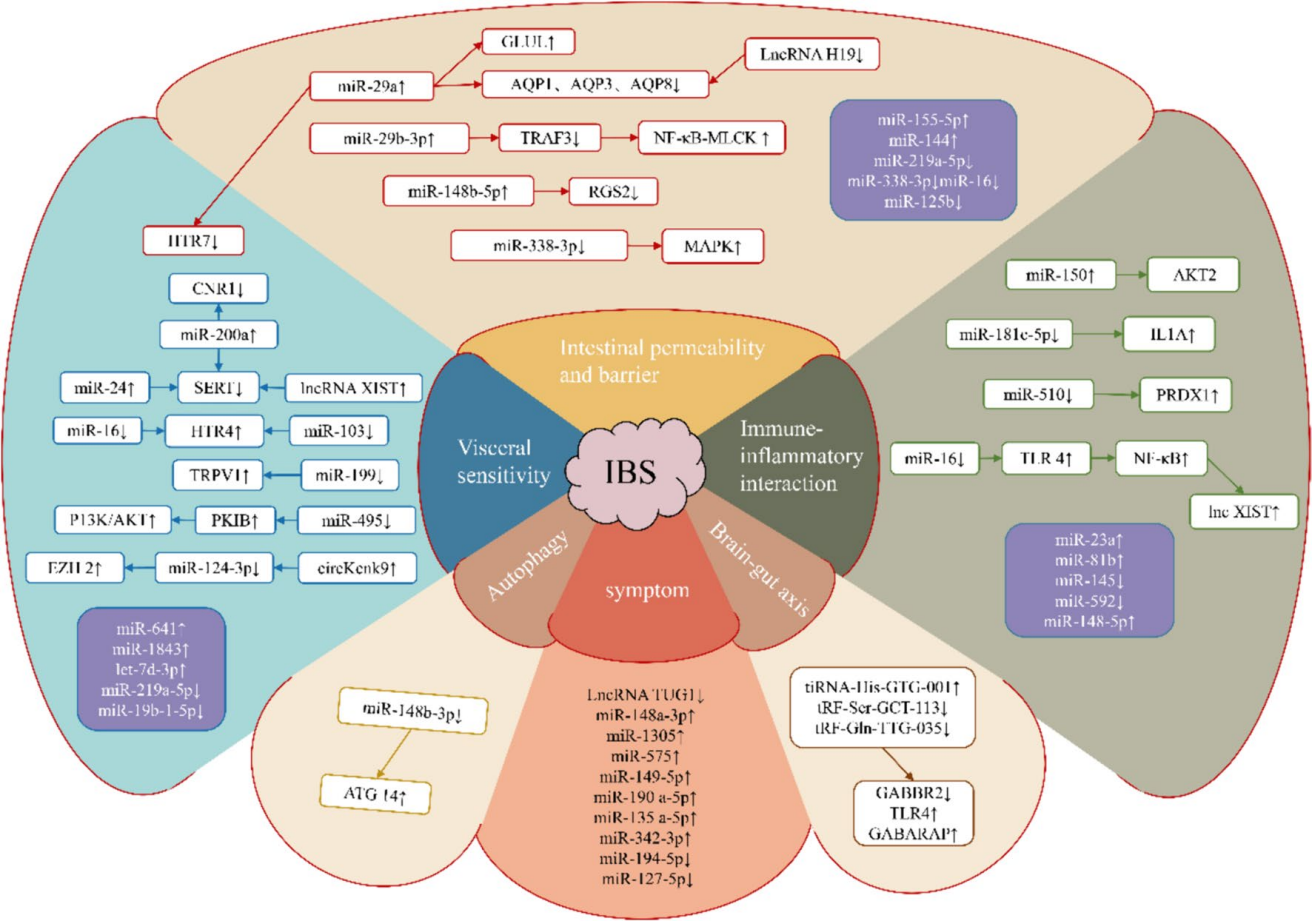
Expression and regulation of target genes of ncRNA in IBS. miR microRNA, LncRNA long non-coding RNA, tiRNA tRNA halves, tRF tRNA-related fragment, circRNA circular RNA, GLUL glutamine synthetase gene, AQP aquaporin, TRAF3 TNF receptor-associated factor 3, MLCK myosin light-chain kinase, RGS2 regulator of G protein signaling 2, MAPK mitogen-activated protein kinase, HTR serotonin receptor, CNR1 cannabinoid receptor 1, SERT serotonin reuptake transporter, PKI protein kinase inhibitor peptide, IL1A interleukin 1α, PRDX1 peroxiredoxin 1, ATG 14 autophagy-related 14, TRPV1 transient receptor potential cation channels 1, TLR4 toll-like receptor 4, EZH2 zeste homolog 2. ↑ increase or activate, ↓ decrease or inhibit

A total of 34 studies with a high correlation between ncRNAs and IBS were selected by reading the included references individually. The studies involved 39 miRNAs, four lncRNAs, three tiRNAs, and one circRNA. Research on miRNAs in IBS is currently a hot topic. Of course, the interactions between miRNAs, lncRNAs, and circRNAs are gradually being revealed. The pathological mechanisms of IBS are mainly related to increased intestinal permeability and mucosal barrier function impairment, increased VH, immunity and low-grade inflammation persistence, the brain-intestinal axis, excessive energy metabolism-associated autophagy, and other factors such as decreased cell viability and gastrointestinal motility. The abnormal expression of ncRNAs in IBS and changes in monosaccharide nucleotide polymorphisms can affect the symptoms of IBS to some extent. Next, the correlation between the pathophysiological mechanisms of IBS and ncRNAs (partially miRNAs) is discussed in specific subsections, and the involvement of ncRNAs in the regulation of IBS pathology is mapped, as shown in Fig. 7.

### VH and ncRNA

VH is one of the main pathological mechanisms of IBS and is the basic pathology of IBS abdominal pain. 5-HT is upregulated in response to visceral pain perception and is considered an indicator of VH [8, 34]. Evidence suggests that 5-HT and 5-HT selective reuptake protein (SERT) are involved in the pathogenesis of IBS [35]. Dysregulation of SERT, which leads to increased mucosal 5-HT availability, may result in high levels of intestinal secretion and motility, potentially hastening the development of IBS-D [36]. A previous study confirmed that miR-24 is upregulated and SERT is reduced in both patients with IBS and rats, and the inhibition of miR-24 has been linked to increased colonic pain and injurious thresholds [37], supporting its involvement in VH. In addition, Hou et al. [30] reported that miR-200a is significantly upregulated in IBS-D rats, accompanied by the downregulation of CNR1 and SERT, suggesting a potential role for miR-200a in modulating the VH response. Zhu et al. [38] observed that miR-29a targets the 5-HT_7_ receptor (HTR7), being significantly upregulated, whereas HTR7 was downregulated in patients with IBS compared with healthy individuals, with similar findings in water-avoidance stress-induced IBS mice. miR-29a knockdown resulted in increased HTR7 expression and reduced VH [38]. Wohlfarth et al. [39] first demonstrated that miRNAs fine-tune the expression of the 5-HT4 receptor (HTR4), and this regulation is affected by the HTR4 polymorphic variant SNPc. *61T > C or low miR-16 and miR-103 expression, suggesting that these miRNAs targeting HTR4 may be involved in IBS-D. Additionally, lncRNA X-inactive specific transcript (lncRNA XIST), a potential oncogene in colorectal cancer, has been identified as a target of SERT through methylation regulation. High lncRNA XIST expression in IBS-D mice promotes SERT promoter methylation, leading to reduced SERT expression and VH [40].

The expression of transient receptor potential vanilla-like subtype 1 (TPRV1) is closely associated with visceral sensation [41]. Research indicates that miRNAs play a critical role in the regulation of TRPV1 [42]. Specifically, miR-199 modulates hyperalgesia by targeting pathways associated with TRPV1. Zhou et al. [42] observed a decrease in miR-199a/b and an increase in TRPV1 in patients with IBS-D experiencing visceral pain, confirming the direct interaction between miR-199 and TRPV1. Furthermore, researchers discovered that miR-199 upregulation in the dorsal root ganglia and colon of rats with VH reversed pain and nociception by suppressing TRPV1 signaling in vivo [42]. Subsequent research on acupuncture therapy revealed that acupuncture enhanced miR-199 expression in the colon, reduced TRPV1 activation, and alleviated VH [43]. Protein kinase inhibitor peptide (PKI) B is a target gene of miR-495, and increased expression of miR-495 reduces VH by inhibiting the PI3K/AKT signaling pathway by downregulating PKIB [44]. In IBS-like rats, the expression of hippocampal circKcnk9, a novel circRNA, is significantly increased. The potential target pathway of circKcnk9 is miR124-3p/EZH2. EZH2 is associated with anxiety and pain. The upregulation of circKcnk9 strongly suppresses the activity of miR124-3p, leading to increased expression of the target gene EZH2, which in turn results in VH and anxiety [45].

### Effects of ncRNA on intestinal permeability and barrier function

Increased intestinal permeability and impairment of intestinal mucosal barrier function are commonly observed in IBS. TJ proteins are crucial components of the intestinal mucosal barrier and are frequently used to assess the integrity of barrier function. Both clinical and basic research have demonstrated increased levels of ZO-1, claudin-1, and occludin, along with reduced levels of claudin-2 in individuals with IBS, leading to increased intestinal permeability. The association between ncRNAs and IBS has garnered considerable attention. Studies have shown that miR-155-5p and miR-144 are upregulated, whereas miR-219a-5p, miR-338-3p, miR-16, and miR-125b are downregulated [46]. This dysregulation is accompanied by a decrease in claudin-1, ZO-1, and occludin in IBS, an increase in claudin-2, and a decrease in the adherents junction protein E-cadherin. The above results show that ncRNA participates in regulating the structure of the TJ and affects barrier function in IBS.

Notably, miR-29 is closely associated with the onset and progression of IBS. Upregulated miR-29a/b and the consequent downregulation of targeted NF-κB inhibitor and claudin-1 are the possible key etiological factors contributing to increased intestinal permeability and chronic gastrointestinal symptoms in patients with IBS-D. In contrast, miR-29a/b silencing flips intestinal hyperpermeability, thus ameliorating intestinal barrier damage [33]. Aquaporins (AQP), water channel proteins capable of transporting water or small solute molecules, forming “pores” in cell membranes, have a pro-homeostatic effect on water metabolism disorders [47]. Studies have demonstrated that AQP is involved in water transport and intestinal mucosal barrier composition in the colon and that a decrease in AQP can lead to increased intestinal permeability [48]. IBS-D is a diarrhea-related dysfunction of colon absorption and water metabolism. Researchers have found that the levels of AQP1, AQP3, and AQP8 are reduced in IBS-D, whereas miR-29a is increased. miR-29a regulates AQP expression, with a negative correlation between them [49]. In addition, glutamine (GLUL), a target of miR-29a, directly regulates intestinal barrier function, and silencing GLUL can enhance epithelial permeability, suggesting that miR-29a regulates intestinal permeability in patients with IBS through a GLUL-dependent mechanism [33].

### Crosstalk between inflammation and immunity with ncRNA

The inflammatory response after infection persists, triggering uncomfortable symptoms in patients with IBS, particularly those with IBS-D and PI-IBS. Research indicates a six-fold increase in the risk of IBS development due to chronic low-grade inflammation of the gastrointestinal tract following infection [50]. Studies have revealed a significant increase in the expression of interferon (IFN)-γ, IL-1β, IL-17, and IL-12 and a notable decrease in IL-10 and IL-4 in the intestinal tissue of patients with PI-IBS [51]. This prompted a re-evaluation of the “functional” paradigm of IBS, as the crosstalk between immune activation and low-grade inflammation is becoming prominent in IBS [52]. Toll-like receptor 4 (TLR 4), an inflammatory pathogen recognition receptor, has been implicated in the initiation of intracellular signal transduction via the NF-κB pathway, contributing to inflammatory responses, antiviral defenses, tumor treatment, and immune response regulation [53]. NF-κB has been identified as a crucial player in intestinal inflammation, which leads to the breakdown of TJs and increased permeability [54]. Xi et al. [55] found increased levels of IL-6 and IL-1β in colorectal tissue in both patients and mice with IBS-D. Meanwhile, researchers observed the presence of TLR4, NF-κB p65, and p-NF-κB in an IBS-D model, indicating an inflammatory response. The same researchers also focused on miR-16, which was found to target TLR4 and inhibit the TLR4/NF-κB signaling pathway in both the IBS-D model and the lipopolysaccharide (LPS)-induced cellular experiments. This inhibition leads to reduced levels of inflammatory cytokines and apoptosis, ultimately preserving TJ integrity [55]. Ji et al. [56] discovered that miR-181c-5p targets and inhibits IL1A expression, resulting in increased abdominal withdrawal reflex and Bristol fecal grade in IBS mice. Furthermore, miR-181c-5p overexpression or IL1A silencing led to reduced expression of tumor necrosis factor-α (TNF-α), IL-2, and IL-6. miR-150, which is associated with inflammatory bowel disease (IBD) and pain, is upregulated and interacts with protein kinase (AKT2), which may affect inflammatory pathways [57]. Zhang et al. [58] confirmed that peroxidoreductase 1 (PRDX1) is a direct target of miR-510. miR-510 inhibits inflammation in Caco-2 cells induced by LPS by upregulating PRDX1, and its interaction with the levels of the pro-inflammatory cytokine TNF-α levels showed a negative correlation. These results suggest that miR-510 targets PRDX1 and regulates inflammation in IBS cells.

### Other potential pathologic mechanisms of IBS with ncRNA

According to Rome IV, the latest definition of IBS is a gut–brain interaction disorder [59]. The gut–brain axis has long been a focus of attention as a bridge between the gut and brain. Recent studies have shown that tRNA-derived small RNAs (tsRNAs), a novel type of small non-coding RNAs, are associated with IBS. Biopsy identification of intestinal tissues from patients with IBS revealed that tiRNA-His-GTG-001 was upregulated, and tRF-Ser-GCT-113 and tRF-Gln-TTG-035 were downregulated. These can regulate gut–brain axis signaling molecules, such as GABBR2, HTR2C, SASH1, TLR4, and GABARAP. Moreover, GABBR2, TLR4, and GABARAP are targeted to participate in GABAergic synapses and TNF-α and other signaling pathways, affecting the clinical symptoms of IBS [60].

Autophagy, a highly conserved catabolic pathway, serves as an intracellular homeostatic mechanism to maintain the balance between protein metabolism and energy under both normal and stressful conditions. Recent evidence has emphasized novel autophagy mechanisms in gastrointestinal diseases, especially those associated with chronic gastrointestinal inflammation and dyskinesia [61]. Excessive autophagy has been found to disrupt the intestinal mucosal barrier, triggering the release of inflammatory mediators and xenogeneic antigens that interact with inflammatory cells in the intestinal mucosa. This cascade of events leads to disturbances in visceral sensitivity and gastrointestinal motility and ultimately causes the development of symptoms such as abdominal pain and diarrhea, which are common in IBS [62, 63]. Recent studies have demonstrated that certain miRNAs modulate autophagy through various pathways. For example, miR-100-5p has been shown to promote autophagy and inhibit apoptosis by regulating mTOR (a key regulator of autophagy) [64]. miR-15b-5p and miR-424-5p compete for binding to plasmacytoma variant translocation 1 (PVT1), resulting in the promotion of autophagy and the downregulation of apoptosis [65]. In a study investigating the effect of apigenin on autophagy in human colon epithelial cells derived from patients with IBS, miR-148b-3p was identified as a key regulator targeting autophagy-related 14 (ATG14). ATG14 overexpression in the presence of miR-148b-3p mimics was found to enhance autophagy in Caco-2 cells, underscoring the significance of the miR-148b-3p/ATG14 signaling axis in human colon epithelial cells and IBS [66].

Mitochondrial DNA (mtDNA) is the only genetic material found in the nucleus. Single nucleotide polymorphisms in mtDNA may increase the risk of IBS because familial clustering of IBS often involves mothers and their offspring [67, 68]. ATP, which is necessary for mitochondria to supply cellular energy, is closely associated with mitochondrial dysfunction, which may result from high-energy requirements in nerves, muscles, and inflammatory cells associated with functional gastrointestinal diseases [67]. Previous studies have suggested that mtDNA polymorphisms are associated with IBS-D [69]. For example, Wange et al. [69] found increased polymorphisms in the MT-ATP 6 and 8 genes in the colon and terminal ileum of IBS-D patients compared to controls. In recent years, mitochondrial dysfunction has been shown to contribute to pain perception and chronic pain [70], and some evidence also suggests that abnormal energy metabolism is a possible mechanism leading to IBS [71], but this still needs to be further verified.

## Discussion

### Analysis of basic information

From the perspective of publications, since 2008, research on the correlation between IBS and ncRNA has shown rapid and stable growth, and the fitted curves show a positive development trend. The interdisciplinary nature of ncRNA and IBS publications emphasizes the importance of interdisciplinary collaborations in future research. IBS has garnered worldwide attention as a global public health problem, with notable contributions from countries such as China, the USA, and Germany that have actively engaged in academic exchanges and cooperation. Niesler Beate’ team has contributed immensely to ncRNA and IBS studies, especially the specific regulation of 5-HT receptor subunit variants with miRNAs, pointing out that mutations in 5-HT3E competitively inhibit the binding of miRNA-510 to the 3′-untranslated region (UTR) of 5-HT3E, which is somehow associated with increased IBS risk in women [27]. They also found that miR-16 and miR-125b downregulation was involved in regulating the upregulation of claudin-2 and cingulin in the jejunum to restore intestinal epithelial barrier function [72]. Although Niesler Beate contributed considerably, their collaboration with other teams was low. Therefore, researchers in the same field must enhance future exchanges and cooperation to foster the advancement of scientific research.

### Hot spot and research trend

Based on highly co-cited documents and keyword clusters and emergence, the pathophysiological mechanisms of IBS involve microbial metabolism, VH, barrier function, the gut–brain axis, autophagy, and monosaccharide nucleotide polymorphisms, in which ncRNAs are involved. Therefore, the search for relevant ncRNA biomarkers as diagnostic and therapeutic candidates for IBS is a hot topic. Additionally, keyword emergence and timeline graphs show when the keywords first appeared in the field and how much attention they received. “microRNAs” had the highest burst intensity at 2.81. miRNAs are a class of RNA oligomers approximately 20–22 bases long that are single-stranded, non-coding RNAs that act as post-transcriptional regulators of gene expression by binding to complementary mRNAs on specific sequences, e.g., the 3′-UTR of target mRNAs, inducing cleavage or translational barriers to regulate target gene expression [73]. Approximately 60% of protein-coding in humans is regulated by miRNAs, and their expression changes can lead to various pathological developments [74–76], which is considered a candidate drug strategy for the diagnosis and treatment of various diseases [77–79]. miRNAs and gastrointestinal diseases, including IBS, exhibit considerable association. In addition, the identification of IBS in other diseases is an important topic. Incidentally, “inflammatory bowel disease” and “cancer” both displayed strong emergence intensity in keyword emergence. The early symptoms of bowel cancer are similar to those of IBS. IBS reportedly frequently co-exists with various gastrointestinal diseases, such as functional dyspepsia and IBD, sharing common clinical symptoms such as abdominal discomfort and pain. The diagnosis of IBS is often based on colonoscopy to exclude organic lesions. However, most patients are unwilling to bear the pain caused by colonoscopy. As a result, the clinical diagnosis of IBS is based on patient symptoms and fecal characteristics, which cannot achieve accurate diagnosis and treatment. Therefore, the development of precise biomarkers for IBS is crucial for accurate diagnosis and treatment.

### ncRNA as a potential strategy for the diagnosis and treatment of IBS

As upstream regulators, ncRNAs have potentially important biological significance in IBS initiation, progression, diagnosis, and treatment. The potential advantages of ncRNAs, especially miRNAs, as novel IBS biomarkers are being actively explored. Previous studies have identified miRNAs such as miR-29a/b, miR-24, miR-510, miR-212, miR-150, miR-342-3p, miR-199a, miR-125b-5p, miR-16, miR-144, miR-200a, miR-214, and miR-103 as potential therapeutic targets for IBS [80, 81]. Simultaneously, some differentially expressed miRNAs have contributed to the diagnosis of different IBS subtypes [82]. Variations in transcriptomic profiles based on mRNA and miRNA may help distinguish UC from IBS [82]. Additionally, miRNA therapy has several advantages. For instance, studies have shown that multiple miRNAs have multiple regulatory mechanisms and are involved in multiple epigenetic events in IBS [83].

In recent years, significant advancements have been made in microbiology, with emerging evidence indicating that small intestinal bacterial overgrowth and microbial dysbiosis play roles in the pathological mechanisms of IBS [84, 85]. The concept of microbiota-gut-brain has received considerable attention in IBS. Consequently, evidence for guided therapies based on the IBS microbiome, such as probiotics, biostimulants, antibiotics, diet, and fecal microbiota transplantation, has become increasingly prominent [86–88]. The imbalance of intestinal flora is not only a pathological result of IBS but also a pathogenic factor. A study has found that specific changes in the composition of the intestinal microbiome, particularly variations in *Bacteroidetes* and *Firmicutes*, are key indicators of ecological imbalance in IBS-D [89]. Previous studies have shown that various miRNAs, including miR-223, miR-155, miR-150, and miR-143, are involved in miRNA-microbiota interactions in the gut [90]. Notably, these miRNAs and others, such as miR-18a*, miR-4802, miR-515-5p, and miR-1226-5p, shape the composition of gut microbiota, ultimately affecting the pathophysiology of intestinal disorders in the host [91]. Olyaiee et al. [92] tested the serum and feces of patients with IBS and found that miR-16 was downregulated, and the relative abundance of *Thickettsia*, *Actinobacteria*, and *Enterococcus faecalis* increased. Mansour et al. [93] found that the number of coliform bacteria increased in patients with IBS, while miR-199b expression decreased, particularly in patients with IBS-D, and intestinal flora negatively correlated with miR-199b. Therefore, exploring the relationship between gut microbiota and miRNAs in IBS is beneficial for clarifying the pathological mechanism of IBS and developing novel therapeutic agents that may bring new hope for the diagnosis and treatment of IBS.

## Strengths and limitations

As a chronic gastrointestinal disorder that affects several individuals, IBS places a heavy burden on healthcare. Despite rapid growth in IBS-related research since Rome I, the diagnosis, pathophysiology, and treatment of IBS remain unclear. In recent years, studies on ncRNAs have shown that they are potential IBS biomarkers that can guide the diagnosis and treatment of IBS. However, the current status and trends of ncRNA studies in IBS have not been comprehensively analyzed. The present study is the first to use bibliometrics to analyze the developmental changes and hotspots of ncRNAs in IBS, focusing on the correlation between miRNAs and the pathological mechanism of IBS and its research trends, although other types of npcRNA were involved, but the literature was very few. This study had some limitations. First, the study may have obtained incomplete literature because of the limited search capacity of WoS, but it is worth noting that WoS is the most widely used scientometrics database, and visualization-based bibliometric analysis lays a foundation for researchers to quickly understand the hotspots and trends in a field. Second, the setting of the retrieval strategy does not guarantee that the included literature is completely consistent with the research topic. Earlier literature has a higher citation rate, and the latest achievements may have insufficient attention due to their late emergence time or inaccurate assessment of the degree of cooperation and contribution of institutions and researchers due to the movement of authors to different work units, among others. Therefore, a bibliometric analysis requires a dialectical view of the advantages and limitations of this method.

## Conclusion

Based on the bibliometric software VOSviewer and CiteSpace, a visual analysis of ncRNAs in IBS was conducted. The main research areas of ncRNAs in IBS include the potential pathological mechanism of IBS, the search for potential ncRNA biomarkers for the diagnosis and treatment of IBS, and the mechanism and biological basis of ncRNA regulation of IBS. To our knowledge, this is the first bibliometric analysis to focus on ncRNAs in IBS, undoubtedly representing an intuitive and practical approach for new researchers who want to quickly understand the status and trends of research in the field or find partners. At the same time, the results of this study also suggest that finding and verifying the internal targets of ncRNAs in IBS are only the first step in the study of their interactions. The realization of clinical transformation to contribute to clinical practice by improving the diagnosis and treatment of IBS remains in the future.

## Supplementary Information

Below is the link to the electronic supplementary material.

Supplementary file1 (XLSX 597 KB)

## Data Availability

The original contributions presented in the study are included in the article/Supplementary material; further inquiries can be directed to the corresponding authors.
